# FROM PAST TO PRESENT: REFINING LONG-TERM CARE POLICIES IN RESPONSE TO CHINA’S AGING POPULATION

**DOI:** 10.1093/geroni/igae098.3177

**Published:** 2024-12-31

**Authors:** Qingwei Wang, Yu Ma, Jiayu Chen

**Affiliations:** Xi’an Jiaotong-Liverpool University, Suzhou, Jiangsu, China; University of Bristol, Bristol, England, United Kingdom; Cambridge University, Cambridge, England, United Kingdom

## Abstract

The evolution of long-term care policy in China is a complex response to the nation’s demographic shift and economic changes. Despite extensive policy development, discrepancies between policy intentions and real-world demands of older adults persist. This study employs a longitudinal analysis to dissect the dynamics that have shaped China’s aging policies from the 1940s to 2023. By critically examining legislative documents and top-level directives, this research identifies gaps between policy formulations and market response and the challenges of navigating a fragmented regulatory landscape. A misalignment has emerged between the supply of Home and Community-Based Services and institutional care demand, resulting in resource underutilization and diverging from intended policy outcomes. The research further explores the regulatory fragmentation within China’s long-term care sector, indicating that decentralized supervision is a significant barrier to achieving cohesive governance and effective policy implementation. The findings illustrate the critical need for adaptive policy mechanisms that better align with demographic realities and consumer expectations. This paper contributes to the discourses on long-term care policy by offering insights into the effectiveness of China’s strategic initiatives in recent years and suggesting directions for future policy refinement. It aims to ensure sustainable care by addressing both the discrepancies in policy and market response and the challenges of fragmented authority.

